# Modelling risk using Bayes theorem of infection by antibiotic-resistant *Escherichia coli* in rural and urban populations of Ecuador

**DOI:** 10.12688/f1000research.14356.1

**Published:** 2018-03-26

**Authors:** Lenin Javier Ramirez Cando, Bence Mátyás, Zayda Jaqueline Lozano-Haro

**Affiliations:** 1 Grupo de Investigación Ambiental para el Desarrollo Sustentable (GIADES), Universidad Politécnica Salesiana, Quito, Ecuador; 2 Grupo de Investigación Mentoria y Gestión del Cambio, Universidad Politécnica Salesiana, Cuenca, Ecuador; 3 Grupo de Investigación en Ciencias Ambientales, Universidad Politécnica Salesiana, Quito, Ecuador

**Keywords:** Antibiotic resistance, Emerging pollutants, Risk assessment

## Abstract

Strains of antibiotic-resistant bacteria have become more and more prevalent. This has attracted the attention of health agencies worldwide, leading to an urgent search for mechanisms to put a stop to this phenomenon. This study focuses on estimating the probability of a person in Ecuador (at potential risk) contracting an infection due to ampicillin-resistant *Escherichia coli* through the consumption of contaminated water, for which a residence area of people was considered in urban or rural areas. The analysis was carried out using the Bayes Theorem and the results show that in the rural population the probability of contracting an infection of this kind is 8.41% whilst in the urban area the probability is 3.57%. These results show an urgent need to provide safe water sources to the population, as well as to instigate an environmental legislation reform that allows for controlling the release of emerging pollutants, including antibiotics.

## Introduction

For years, humankind has sought to mitigate the impacts produced by human development. Perhaps one of the most serious and unregulated problems in legislation is that of emerging pollutants (EP). According to Fernández *et al.* (2016) ^1^ these are common-use chemical compounds of natural or synthetic origin, which, despite not being considered significant in terms of distribution and/or concentration, present a risk to the environment and human health. Within EPs, antibiotics make up a group that is becoming increasingly important and their presence in the environment has become a global public health problem. Concern surrounding the presence of antibiotics in bodies of water and the subsequent increase in bacterial resistance has led the WHO to publish a list of bacteria that have developed capacities for inhibiting widely-used antibiotics. Bacterial resistance according to Rodríguez, A. (2016) ^2^ arises from the excessive and irrational use of antibiotics to treat infections that affect human beings’ bodies. This is due to an independent adaptive selection of the bacteria and the family of a specific strain as in the case of *Staphylococcus aureus* and its ability to inhibit conventional penicillin. This is not an isolated event; on the contrary, Cabrera, C. *et al.* (2007) ^3^ maintain that by mutations in the chromosome of certain bacteria or by genetic exchange, bacteria can develop high innate resistance to antibiotics with several mechanisms between species of the same family or between different families. This study will explore the relationship that exists between bacterial resistance to antibiotics as a problem of emerging pollutants and public health for the inhabitants of Ecuador, taking area of residence as the reference.

## Methods

In order to estimate the probability of an Ecuadorian contracting an antibiotic-resistant bacterial infection, one must consider the area where the person lives, the presence or absence of *Escherichia coli* in the main sources of water for human consumption in the country, and the antibiotic to be analysed (in this case ampicillin). The analysis was carried out considering the Bayes Theorem which, according to Marrero, D. (2014) ^4^, is expressed as the conditional probability of a random event ‘A’ given another event ‘B’. Below is its formula, which takes into account that several events of A can be exclusive:

$$P(A_{i}|B=\frac{P(B|A_{i})P(A_{i})}{\sum_{i=1}^{k}P(B|A_{i})P(A_{i})}$$

Where: *P*( *A _i_* ) are the *a priori* probabilities.

*P*( *B|A _i_* ) are the probabilities of B in hypothesis *A _i_*.

*P*( *A _i_ |B*) are the *a posteriori* probabilities.

$\sum_{i=1}^{k}P(B|A_{i})P(A_{i})$ is the sum of the probabilities of B in the hypothesis *Ai* times the *a priori* probabilities.

Our analysis focuses on the occurrence of three events:

- (i) the probability of consuming contaminated water, depending on the area where a person lives.
- ii) the probability that a person who consumes contaminated water will contract an infection due to *Escherichia coli*.
- iii) the probability that the contracted infection is resistant to antibiotics, using ampicillin as a reference.

## Results

### Determination of the probability that an Ecuadorian will consume contaminated water according to the area where the person lives

In order to carry out this estimate, data published by the INEN according to Ecuadorians’ areas of residence has been taken as a reference. The data are shown in Table 1:

**Table 1.** Urban and rural population in Ecuador, 2010. //Source: INEC, Population census (2010) ^5^.

Area	Population	%
**Urban**	9,090,786	63%
**Rural**	5,392,713	37%
**Total**	14,483,499	100%

In the same way, the INEN provides information regarding water quality (shown in Table 2):

**Table 2.** Water quality according to area of residence. Source: INEC Survey (December, 2016) ^6^.

Area	% of uncontaminated water consumption	% of contaminated water consumption
**Urban**	84.6	15.4
**Rural**	68.2	31.8
**Total**	100	100

Thereafter, we applied the aforementioned Bayes Theorem to find out the probability that people living in urban or rural areas who consume drinking water may be contaminated with *Escherichia coli*. For this case:

*P*( *A _i_ |B*) is *P*( *urban|contaminated water*)

*P*( *A _i_* ) is *P*( *urban*)& *P*( *rural*)

*P*( *B|A _i_* ) is *P*( *contaminated water|urban*)&

*P*( *contaminated water|rural*)

$$\begin{array}{c} \;P(urban|contaminatedwater)=\\ \frac{P(contaminatedwater|urban)}{\sum_{i=1}^{k}P(contaminatedwater|urban,rutal)P(urban,rutal)} \end{array}$$

P(urban|contaminatedwater)=45.19%

By carrying out the same analysis for the rural population, one obtains a 54.81% probability that a person living in the rural area may consume contaminated water ( Table 3).

**Table 3.** Summary of the probability calculation for the different events

	Distribution of people living in urban or rural areas	Probability of consuming contaminated water	Probability of contracting an *E. coli* infection	Probability of contracting an infection with a resistant strain	Probability of contracting an ampicillin-resistant infection due to contaminated water consumption
**People living in** **urban areas**	63 %	45.19 %	38.08 %	32.97 %	3.57%
**People living in rural** **areas**	37 %	54.81 %	61.92 %	67.03 %	8.41%

### Determination of the probability that an Ecuadorian who consumes contaminated water will contract an infection due to *Escherichia coli*

Once the probability of a person drinking contaminated water is known, it is necessary to find out the probability of contracting an infection due to Escherichia coli by consuming contaminated water. According to Vila, J. *et al*.(2016) ^7^, in a study of 33 people living in South America in urban and rural areas, there is a 9.1% and 12.2% respectively that they house the aforementioned bacteria.

### Determination of the probability that an Ecuadorian who consumes contaminated water will contract an infection caused by antibiotic-resistant *Escherichia coli*

Once the probability of contracting an *E. coli* infection due to contaminated water consumption is identified, it is necessary to find out how many of these infections are resistant to antibiotics, which in our case was ampicillin. Bianchi, V. *et al*., (2014) ^8^, in a study conducted in the San Juan River in Argentina, showed that the average ampicillin-resistant UFC percentage in urban areas was 73.39% and for rural areas 92.85%. Using the Bayes Theorem for each of the cases described above, we obtained the following results:

## Conclusions

This study shows that both urban and rural populations are exposed to an antibiotic-resistant infection. However, Ecuador’s rural population is more exposed because the water sources they use are not safe. This draws attention to the necessity of providing safe, clean drinking water to the entire population. Even so, the high standards of water quality that many Ecuadorian cities have does not completely eliminate the risk of contracting antibiotic-resistant infections, thus demonstrating that an urgent legislation reform is required in order to control the release of these types of pollutants into bodies of water.

## Data availability

Population and water quality data was obtained from the Instituto Nacional de Estadística y Censos (INEC), Population census (2010): http://www.ecuadorencifras.gob.ec/base-de-datos-censo-de-poblacion-y-vivienda/ and INEC, Survey (2016): http://www.ecuadorencifras.gob.ec/documentos/web-inec/EMPLEO/2017/Indicadores%20ODS% 20Agua,%20Saneamiento%20e%20Higiene/ Presentacion_Agua_2017_05.pdf
