# Supplementary material for: Modelling risk using Bayes theorem of infection by antibiotic-resistant Escherichia coli in rural and urban populations of Ecuador
Source: F1000Res. 2018 Mar 26;7:375. [Version 1] doi: 10.12688/f1000research.14356.1 (PMC6509746; doi:10.12688/f1000research.14356.1)
Supplement: Peer review history

# Peer review: Reviewer response for version 1

**DOI:** 10.5256/f1000research.15619.r47288

**Published:** 2019-05-08

**Authors:** Imre Vágó

**Affiliations:** 1 Institute of Agricultural Chemistry and Soil Science, Faculty of Agricultural and Food Sciences and Environmental Management, University of Debrecen, Debrecen, Hungary

I read with great interest Ramirez and colleagues’ study in which they present an estimation about the probability of contracting infection due to ampicillin-resistant *E. coli* though the consumption of contaminated water in the urban and rural areas of Ecuador.

 This publication is a good example for how to obtain valuable and useful knowledge through consistent data analysing and data evaluation. The goal of the study is well described. The manuscript is clear; the used methods in this paper are adequate. However, I would like to share my observations in order to aid readers’ understanding.

- Despite the authors analysing separately the urban and rural populations of Ecuador, the basis of the differentiation is not defined.
- In this paper, the data of urban and rural populations in Ecuador is outdated; it referred to 2010 data (Table 1). I mean, it would be better to use the latest published dataset from 2018, source: https://tradingeconomics.com/ecuador/rural-population-percent-of-total-population-wb-data.html. It states that the total population is 16,624,858 and also the ratio of urban population (64.3%) increased, compared to 2010.
- The ratio of population consuming *Escherichia coli*-infected water (Table 2) is surprisingly high, especially for the rural population, compared to Europe. Does it really cover reality? Using ampicillin resistance values measured in the Bianchi *et al.* (2014) study in the water of San Juan River in Argentina, is critical. The distance is about 3600 km, the climate conditions are significantly different. Sorry, I am not aware of the ampicillin application frequency in South America, perhaps this is the reason for my doubts about this data.
- Last but not least: there are two typos in the paper. One of them is the missing closing bracket “)” before the equal sign ‘=’ in the Marrero’s equation. The other error is in Table 2; instead of last row (Total, 100, 100) it would be better to create another column, with the identical name and values.

In conclusion, the results of the research work are proving that the Bayes Theorem is suitable for the evaluation of elaborated data sets. The publication meets all of the scientific requirements; the results have practical relevance and they can be used by the decision makers also. That is why I suggest the presented paper for indexing.

I have read this submission. I believe that I have an appropriate level of expertise to confirm that it is of an acceptable scientific standard.

# Peer review: Reviewer response for version 1

**DOI:** 10.5256/f1000research.15619.r32439

**Published:** 2018-05-08

**Authors:** Andrés Ricardo Izquierdo Romero

**Affiliations:** 1 Centro de Nanociencias y Nanotecnología; Departamento de Ciencias de la Vida; Grupo de Investigación en Microbiología y Ambiente GIMA, Universidad de las Fuerzas Armadas ESPE, Sangolquí, Ecuador

The manuscript is clear, however in the manuscript is cited Fernández et al. (2016), and it is not found in the bibliography. I recommend placing more citations of other investigations where Bayes Theorem is used, for this type of investigation. 

 The statistical analysis if applicable, however I recommend placing the calculations to obtain the values ​​that were obtained and discussed, such as:

- Probability of contracting an *E. coli *infection: 38.08% (people living in urban areas), 61.92% (people living in rural areas)
- Probability of contracting an infection with a resistant strain: 32.97% (people living in urban areas), 67.03% (people living in rural areas)
- Probability of contracting an infection resistant to ampicillin due to the consumption of contaminated water: 3.57% (people living in urban areas), 8.41% (people living in rural areas)

I suggest that it is necessary to show the data for water pollution in rural and urban areas of Ecuador, as well as the parameters for water quality according to the area of ​​residence, such as water contamination with ampicillin-resistant *E. coli,* for estimate the probability of a person in Ecuador contracting an ampicillin-resistant *Escherichia coli *infection through the consumption of contaminated water, as the manuscript suggests.

 The data of Vila, J. et al. (2016) can be used statistically, is a small study of 33 people living in South America in urban and rural areas, compared to the urban and rural population in Ecuador, 2010, which is 14,483,499 people, for the determination of the probability that it is Ecuadorian to consume contaminated water. an infection by Escherichia coli. There are data on *E. coli i*nfections in contaminated water in Ecuador, to strengthen the results obtained.

 To determine the probability that an Ecuadorian will consume contaminated water to contract an infection caused by antibiotic-resistant Escherichia coli, used data from Bianchi et al. (2014) of the San Juan River in Argentina, showing that the average percentage of CFU resistant to ampicillin in urban areas was 73.39% and for rural areas 92.85%. These data are comparable with water sources in Ecuador, such as rivers, environmental conditions, etc., to be able to use them. Similarly, it is suggested to know if there are data of E. coli infections resistant to antibiotics in contaminated water in Ecuador.

 In conclusion, the objective of the work is interesting, however, he suggested that to investigate more real data of Ecuador, to strengthen the obtained results.

I have read this submission. I believe that I have an appropriate level of expertise to confirm that it is of an acceptable scientific standard, however I have significant reservations, as outlined above.

# Response

**Published:** 2018-12-11

**Authors:** Bence Mátyás

**Affiliations:** Universidad Politécnica Salesiana, Ecuador

Dear Dr. Andrés Ricardo Izquierdo Romero, thank you for your report. 

*"The manuscript is clear, however in the manuscript is cited Fernández et al. (2016), and it is not found in the bibliography. I recommend placing more citations of other investigations where Bayes Theorem is used, for this type of investigation."*

authors' reply:

We will ask for a minor correction in the article regarding the missing citation. Considering this piece is a research note, we do not think that expanding the article with more citations is necessary.

*"I suggest that it is necessary to show the data for water pollution in rural and urban areas of Ecuador, as well as the parameters for water quality according to the area of residence, such as water contamination with ampicillin-resistant E. coli, for estimate the probability of a person in Ecuador contracting an ampicillin-resistant Escherichia coli infection through the consumption of contaminated water, as the manuscript suggests."*

authors' reply:

This information is presented in external links only in order to keep the research note brief.

*"The data of Vila, J. et al. (2016) can be used statistically, is a small study of 33 people living in South America in urban and rural areas, compared to the urban and rural population in Ecuador, 2010, which is 14,483,499 people, for the determination of the probability that it is Ecuadorian to consume contaminated water. an infection by Escherichia coli. There are data on E. coli infections in contaminated water in Ecuador, to strengthen the results obtained."*

authors' reply:

The Bayes theorem allows to add more specific information later in a new analysis.

*"To determine the probability that an Ecuadorian will consume contaminated water to contract an infection caused by antibiotic-resistant Escherichia coli, used data from Bianchi et al. (2014) of the San Juan River in Argentina, showing that the average percentage of CFU resistant to ampicillin in urban areas was 73.39% and for rural areas 92.85%. These data are comparable with water sources in Ecuador, such as rivers, environmental conditions, etc., to be able to use them. Similarly, it is suggested to know if there are data of E. coli infections resistant to antibiotics in contaminated water in Ecuador... *In conclusion, the objective of the work is interesting, however, he suggested that to investigate more real data of Ecuador, to strengthen the obtained results."

authors' reply:

The Bayes theorem allows to add the specific information later in a new analysis, making analysis deeper, however, this research note tends to offer the first step for a further analysis regarding Bayesian statistics.

# Peer review: Reviewer response for version 1

**DOI:** 10.5256/f1000research.15619.r32440

**Published:** 2018-03-28

**Authors:** Ramirez Robles Jorge Yandry

**Affiliations:** 1 Departamento de Química y Ciencias Exactas, Universidad Técnica Particular de Loja, Loja, Ecuador

The methods in this paper are adequate, however, in probability theory, according to Bayes’ theorem that describes the probability of an event, based on prior knowledge of conditions that might be related to the event. So, in case of this paper I think it is necessary to show data about pollution water made in different areas of Ecuador, for two reason: the first one is to make known the difference between rural and urban area, and the second one, if “ *this study focused on estimating the probability of a person in Ecuador contracting an infection due to ampicillin-resistant Escherichia coli through the consumption of contaminated water”*, it’s necessary to know if the source of water in Ecuador are contaminated with ampicillin-resistant *E. coli*. So, if the water in Ecuador is contaminated with ampicillin-resistant *E. coli*, is highly probably that the people of Ecuador contracting an infection, then using Bayes’ theorem, a water quality of water (contaminated with E. coli) can be used to more accurately assess the probability that the contracting an infection, compared to the assessment of the probability of people contracting infection made without knowledge of data of quality of water.

 In conclusion the paper is correct because the aim is shows that both urban and rural populations are exposed to an antibiotic-resistant infection, and in Ecuador there aren't researched in this area. For this reason I think the worked can be published.

I have read this submission. I believe that I have an appropriate level of expertise to confirm that it is of an acceptable scientific standard.

# Response

**Published:** 2018-12-11

**Authors:** Bence Mátyás

**Affiliations:** Universidad Politécnica Salesiana, Ecuador

We are grateful for Dr. Ramirez Robles Jorge Yandry's report,

We just wish to add that considering Bayes theorem capacity to aggregate new information, starting with non-known data analysis is an effective way.
